# Daily Usage of Proton Pump Inhibitors May Reduce the Severity of Critical Upper Gastrointestinal Bleeding in Elderly Patients

**DOI:** 10.1155/2020/7168621

**Published:** 2020-08-06

**Authors:** Hidetaka Matsuda, Takuto Nosaka, Yu Akazawa, Yasushi Saito, Yoshihiko Ozaki, Kazuto Takahashi, Tatsushi Naito, Kazuya Ofuji, Masahiro Ohtani, Katsushi Hiramatsu, Yasunari Nakamoto

**Affiliations:** Second Department of Internal Medicine, Faculty of Medical Sciences, University of Fukui, Fukui, Japan

## Abstract

**Introduction:**

We retrospectively examined the relationship between daily proton pump inhibitor (PPI) use and severity of upper gastrointestinal bleeding (UGIB), mainly in the elderly.

**Methods:**

We included 97 patients with nonvariceal UGIB diagnosed at our hospital from January 2012 to October 2017. Bleeding severity was assessed using the shock index (SI) and estimated bleeding volume; 49 patients met the criterion for the mild group and 48 for the moderate/severe group. The effect of PPI use on bleeding severity was compared between the groups. The relationships of PPI use and dose with the clinical symptoms of UGIB were also analyzed.

**Results:**

Among the 97 patients, 17 (17.5%) habitually used PPIs. The rate of habitual PPI use was significantly higher in the mild group, indicating as an independent factor contributing to a reduction in the severity of UGIB in a multiple logistic regression analysis (30.6% vs. 4.2%; OR 10.147; 95% CI 2.174–47.358, *P* < 0.01). When analyzing data for a subgroup of patients older than 75 years, we found the protective PPI effect to be even higher in the mild UGIB group than in the moderate/severe group (37.0% vs. 5.6%; OR 10.000; 95% CI 1.150–86.951, *P* < 0.05). Conversely, we found no association between PPI prescription and UGIB symptoms in patients younger than 75 years. The mean estimated bleeding volume and SI in the 17 habitual PPI users were both significantly less than those among the 80 nonhabitual users, respectively (*P* < 0.05). The proportion of patients with mild UGIB was similar between the low- and high-dose PPI users.

**Conclusions:**

Particularly in elderly patients with nonvariceal UGIB, habitual PPI use can alleviate the clinical symptoms of UGIB by suppressing the volume of bleeding, regardless of the adapted dose of PPIs.

## 1. Introduction

Upper gastrointestinal bleeding (UGIB) is a serious condition that can take a severe course [1, 2]. UGIB can be fatal, particularly in elderly patients, whose physical strength has declined over time and who have various underlying diseases [3–7]. Currently, populations, primarily in developed countries, are rapidly aging [8], and even in Japan, elderly individuals over the age of 75, comprise approximately 15% of the total population [9]. Therefore, treating UGIB is an important issue that needs to be incorporated into routine medical care, especially among elderly patients [5]. Habitual proton pump inhibitor (PPI) use is known to decrease the incidence rate of UGIB among individuals taking drugs that might induce UGIB (e.g., nonsteroidal anti-inflammatory drugs [NSAIDs], antiplatelet drugs, anticoagulants) [10, 11]. Additionally, the inhibitory effect of these PPIs on UGIB incidence has been reported to be more effective in elderly populations than in others [12, 13]. However, to what extent habitual PPI use influences clinical UGIB symptoms and severity remains unclear. In the present retrospective study, we investigated the influence of habitual PPI use on clinical symptoms and bleeding severity (determined by the shock index (SI) and estimated bleeding volume) in nonvariceal UGIB cases, taking miscellaneous patient characteristics such as age into account, to formulate countermeasures for UGIB in an aging society.

## 2. Methods

### 2.1. Subjects

We retrospectively analyzed anonymous clinical data of 97 patients with nonvariceal UGIB diagnosed by upper gastrointestinal endoscopy at our hospital from January 2012 to October 2017 (mean age 71.6 ± 1.3 years, males/females: 62/35 cases). All patients (or their parents or guardians) had agreed to the treatment of UGIB by providing written informed consent. This study was performed in compliance with relevant laws and institutional guidelines and following the ethical standards of the Declaration of Helsinki. This study was approved by the institutional review board (IRB) of the University of Fukui (IRB number: 20170132). In this study, no additional intervention was conducted on the subjects, and informed consent was obtained in the form of opt-out on the website of the University of Fukui Hospital (http://research.hosp.u-fukui.ac.jp/rinsho/).

### 2.2. Definition of Upper Gastrointestinal Bleeding

Using the review of Kamboj et al. [2] as a reference, UGIB was defined as bleeding caused by benign/malignant diseases (ulcers, solid cancers, vascular abnormalities, etc.) in the esophagus, stomach, and duodenum.

### 2.3. Determination of Upper Gastrointestinal Bleeding Severity

The estimated bleeding volume and SI were used as parameters to determine the bleeding severity in each UGIB case. Using the hemoglobin values recorded on the patients' medical records before bleeding and at the time of UGIB diagnosis, we calculated each patient's estimated bleeding volume using Nadler's equation, which can enable assessment of the individual's total blood volume [14]. The SI was calculated by dividing the heart rate by the systolic blood pressure [15].

Using the bleeding severity classifications of the American College of Surgeons and other reports that investigated the cut-off value of the SI in UGIB cases as references [16–18], the present study classified bleeding severity as follows: mild: estimated bleeding volume < 1,000 mL and SI < 1.0; moderate: estimated bleeding volume 1000–2000 mL or SI 1.0–2.0; severe: estimated bleeding volume > 2000 mL or SI > 2.0.

### 2.4. Patient Classification and Analysis Categories

Of the total 97 cases, 49 were classified as mild cases, and 48 were classified as moderate/severe cases according to bleeding severity. The effect of PPI use on bleeding severity was compared and analyzed between the groups, along with the lesions responsible for the bleeding, underlying diseases exacerbated by aging, and patient characteristics selected as risk factors for UGIB (age, sex, underlying disease [hypertension/diabetes/dyslipidemia/cerebrovascular disease/heart disease/hepatic cirrhosis/kidney disease/dementia/extra-gastrointestinal malignant tumors], antithrombotic drugs [antithrombotic drugs/anticoagulants], NSAID use) [1, 2, 7, 19–21]. Additionally, correlations between PPI use/dose and clinical UGIB symptoms (estimated blood volume, SI, total transferred blood volume) were also analyzed.

### 2.5. Statistical Analysis

GraphPad Prism ver. 6.0 (GraphPad Software, Inc., La Jolla, CA, USA) and SPSS Statistics ver. 20 (SPSS Inc., Chicago, IL, USA) were used for statistical analysis. Associations between the severity of UGIB and the clinical characteristics (including the daily usage and administered dose of PPI) of subjects were examined using Fisher's exact probability test for univariate analysis and multivariable logistic regression for multivariable analysis. Welch's *t*-test was used for comparing clinical UGIB symptoms (estimated bleeding volume, SI, total transferred blood volume) between PPI users, and nonusers. *P* < 0.05 was considered significant.

## 3. Results

Results of subject characteristic influences on UGIB severity are shown in Table 1(a). Mean ages in both mild and moderate/severe cases were high, at 72.3 ± 12.2 and 69.9 ± 13.6 years, respectively, with no statistically significant differences between the two groups. There was also no difference between the groups in terms of sex and the compositional ratio of diseases causing UGIB. Most patients had some form of underlying disease (mild group: 81.6%, moderate/severe group: 79.2%, *P* > 0.05). Among all subjects, 17.5% (17/97) used PPI. Habitual PPI use in the mild group (30.6%) was statistically significantly higher (*P* < 0.01) than that in the moderate/severe group (4.2%), and the multiple logistic regression analysis showed that habitual PPI use is a contributing factor to decreased bleeding severity (odds ratio [OR]: 10.147, 95% confidence interval [CI] 2.174–47.358, *P* < 0.01). PPIs were administered in seven cases to prevent the recurrence of peptic ulcer disease, in five cases for combined usage with antithrombotic drugs, in four cases for gastroesophageal reflux disease (GERD), and in one case for gastric cancer. Of those 17 cases, 11 showed concordance between the targeted lesion for PPI administration and the source of bleeding. In terms of the duration of PPI usage, there was no difference between the two groups (proportion of >6 months duration 11/15 vs. 2/2). Correlations between other habitually used drugs and bleeding severity were also analyzed in the present study. No statistically significant differences were observed in H_2_ receptor blocker use between the two groups. Additionally, no statistically significant influence of habitual antiplatelet drug and anticoagulant use, including its complications, was observed on bleeding severity. There was also no difference in *Helicobacter pylori* (*H. pylori*) infection rate between the two groups, 59.3% of the mild group vs. 70.0% of the moderate/severe group.

Subjects were classified into two groups (above and below 75 years old), and factors relating to nonvariceal UGIB severity were investigated in each group (Tables 1(b) and 1(c)). Habitual PPI use in the mild group over the age of 75 (37.0%) was statistically significantly higher (*P* < 0.05) than that in the moderate/severe group (5.6%), and habitual PPI use in elderly individuals was determined as a factor contributing to decreased UGIB severity (OR: 10.000; 95% CI 1.150–86.951, *P* < 0.05). Habitual PPI use in the mild group under the age of 75 also showed higher tendencies than that in the moderate/severe group, but no statistically significant differences were observed (22.7% vs. 3.3%, *P* > 0.05). The present study did not identify other factors relating to bleeding severity in either the elderly (over 75 years) and younger (under 75 years) groups.

**Figure 1:**
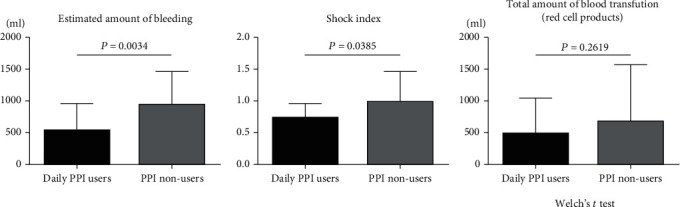
Comparisons of the clinical manifestation of upper gastrointestinal bleeding (estimated amount of bleeding, shock index, and total amount of blood transfusion) between 17 patients with and 80 patients without daily usage of proton pump inhibitors (PPIs). The mean estimated bleeding volume and SI in the habitual PPI users were both significantly less than those among the non-habitual users, respectively (*P* < 0.05). Meanwhile, there was no significant difference in the average volume of packed red blood cell volume transfused while being hospitalized between patients' groups of habitual or nonhabitual PPI users (*P* > 0.05).

The influence of habitual PPI use on clinical manifestations of nonvariceal UGIB was investigated (Figure 1). The average estimated bleeding volume in the 17 subjects who habitually used PPI was 591 ± 109.1 mL, which was statistically significantly less (*P* < 0.05) than that in the nonhabitual users, at 944 ± 62.6 mL. Additionally, the average value of the SI at the time of bleeding in habitual PPI users was statistically significantly lower than that in nonhabitual PPI users (0.73 ± 0.27 vs. 0.98 ± 0.49, *P* < 0.05). Meanwhile, the average volume of packed red blood cell volume transfused while being hospitalized was lower in habitual PPI users than in nonhabitual users, but no statistically significant differences were observed (477.6 ± 566.7 mL vs. 672.0 ± 100.9 mL, *P* > 0.05).

**Figure 2:**
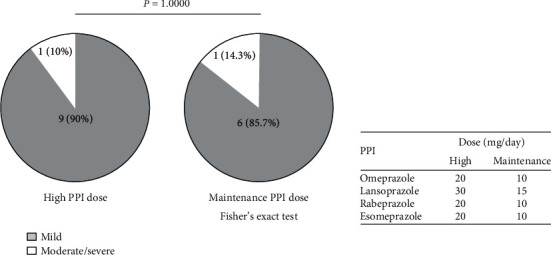
Comparisons of the frequencies of nonvariceal upper gastrointestinal bleeding (UGIB) between 10 patients with high-dose and seven patients with maintenance dose of daily proton pump inhibitor (PPI) consumption. The proportion of patients with mild UGIB was similar between the low- and high-dose PPI users.

Finally, correlations between PPI dose and UGIB severity in the 17 habitual PPI users are shown in Figure 2. Of the seven patients who took a maintenance PPI dose (omeprazole/rabeprazole/esomeprazole: 10 mg/day, lansoprazole: 15 mg/day) before bleeding, 85.7% were mild cases, and of the 10 patients who took a high PPI dose (omeprazole/rabeprazole/esomeprazole: 20 mg/day, lansoprazole: 30 mg/day), 90.0% were mild cases; no statistically significant differences were observed (*P* > 0.05).

## 4. Discussion

In this retrospective analysis, daily usage of PPI was proved to reduce the severity of UGIB, especially in patients older than 75 years old.

PPI inhibits gastric acid secretion and serves as a mucosal treatment for reflux esophagitis and peptic ulcer cases that cause UGIB [22–24]. PPI administration is known to reduce UGIB incidence rates in high-risk UGIB cases [10, 11], and reports have indicated that its preventative effects are particularly high in elderly individuals [12, 13]. However, the extent to which PPI contributes to UGIB severity is unclear. In the present study, we investigated nonvariceal UGIB cases in our hospital and correlations between habitual PPI use and UGIB severity, as well as age and other clinical characteristics.

The bleeding severity of each enrolled subject was determined based on the estimated bleeding volume and SI, and multivariate analysis was used to compare clinical characteristics between mild and moderate/severe cases. Results showed that PPI use is an independent factor that reduces UGIB severity, with a similar effect observed in the case group over the age of 75. In patients under the age of 75, a higher frequency of habitual PPI users was observed in the mild bleeding group than in the moderate/severe group, although the difference did not reach statistical significance. Recently, Li et al. [4] conducted a prospective study that included patients in whom antiplatelet drugs were administered as secondary prevention against the development of cardiovascular events, and they reported that the numbers needed to treat for concomitant PPI use in the prevention of severe UGIB is low in subjects over the age of 75. Results from the present study and those of Li et al. [4] indicate that habitual PPI use in elderly individuals can prevent a worsening general condition that accompanies gastrointestinal bleeding. As far as we know, the present study is the first report that investigated correlations between PPI and UGIB severity.

The present study used the SI and estimated blood volume to investigate the influence of habitual PPI use on UGIB severity. Results showed that PPI users had statistically significantly lower SI and estimated blood volume than nonusers. These results indicate that habitual PPI use can reduce the bleeding severity in UGIB cases by inhibiting total bleeding volume. The Rockall, AIMS65, and Glasgow–Blatchford scores have been reported as useful preendoscopic risk assessment tools [1, 25]. Meanwhile, these require biochemical blood examination results, and calculations are rather complex.

In comparison, SI calculation is simple and suitably reflects circulatory dynamics, with reports indicating that it has a practical use for UGIB identical to that of other scoring methods [15, 26]. The present analysis used the SI and estimated bleeding volume to investigate direct correlations between PPI and blood loss caused by UGIB. Among the 46 patients with hypertension in our present study, only one was using beta-blockers. Thus, we believe that the SI was not influenced by beta-blocker usage in our study and was, therefore, not underestimated.

Reports have indicated that controlling transferred blood volume can result in the reduction of early death and re-bleeding rates in acute UGIB cases [27]. For this reason, it is recommended that the hemoglobin threshold be maintained at 70–80 g/L for UGIB cases [1]. The present investigation did not show statistically significant differences in PPI use concerning the total transferred blood volume related to UGIB. This may have been partly due to appropriate blood transfusion measures by the primary physicians following diagnoses of individual subjects, to some extent.

Some issues must be addressed if the present results are to be applied in the clinical setting. First, we must consider which cases primarily among elderly individuals are suitable for habitual PPI use. As mentioned previously, the elderly population is currently increasing, and administering PPI to all elderly individuals over the age of 75 is not practical. Guidelines recommend maintenance PPI administration for patients with GERD accompanied by sores and high relapse rates of peptic ulcers (e.g., NSAIDs, low-dose aspirin administration cases, cases without *H. pylori* eradication treatment) [24, 28]. It is predicted that PPI use in these types of cases would result in not only the reduction of gastrointestinal bleeding frequency but also bleeding severity. Meanwhile, the extent to which habitual PPI use should be indicated for cases besides these is still up for debate. In this study, 17 patients suffered from UGIB despite the daily usage of PPI. Of note, however, was that the bleeding severity was mild in 15 of the 17 patients. Future studies are required to clarify the characteristics of patients with an extremely high risk of UGIB, such as older adult patients. Habitual PPI usage in such patients can potentially minimize the harmful effects of UGIB.

The next issue involves considering adverse events accompanying long-term PPI administration. Correlation of long-term PPI administration with various adverse events including osteoporosis-related bone fracture, *Clostridium difficile* infection, pneumonia, vitamin B12 deficiency, kidney disease, and dementia has been previously reported, although the findings are controversial [29–32]. In an expert review published by the American Gastroenterological Association, the benefits of appropriate PPI prescriptions are thought to outweigh their risks; the authors add that there is insufficient evidence to recommend specific strategies for mitigating the adverse effects of PPI usage, including long-term PPI usage [31]. Nevertheless, maintenance with the minimum dose necessary is recommended when administering PPI over a long period, especially in older adults [24, 29, 31]. The present results, which showed that habitual PPI use at a maintenance dose resulted in decreases in UGIB severity, will pave the way for the development of PPI administration methods that minimize adverse event risks without negatively affecting the patient's quality of life.

The mechanisms by which habitual PPI use reduced both the incidence and severity of UGIB should be investigated further. PPI has been shown to reduce the severity of gastrointestinal mucosal injury by suppressing gastric acid secretion [33]. In addition, since most UGIB is caused by damage of the gastrointestinal mucosa [2], it is expected that the severity of UGIB will be reduced in habitual PPI users, most likely due to constant suppression of gastric acid secretion [34, 35].

## 5. Conclusions

Habitual PPI use was shown to diminish clinical symptoms by reducing bleeding volume in nonvariceal UGIB cases, particularly among elderly individuals. Additionally, even low doses of habitual PPI were shown to possibly reduce UGIB severity. Further investigations are required to establish a suitable PPI administration method to alleviate UGIB symptoms in elderly individuals.

## Figures and Tables

**Table 1a:** (a) Clinical characteristics of all 97 patients enrolled in this study

Characteristics	Bleeding severity		Univariate *P* value	Multivariate	
	Mild *n* = 49 (%)	Moderate/severe *n* = 48 (%)		OR (95% CI)	*P* value
Age	72.3 ± 12.2	69.9 ± 13.6	0.2174	—	n.s.
Male	31 (63.3)	31 (64.6)	1.0000		
Etiology of bleeding					
Esophageal ulcer	2 (4.1)	2 (4.2)	1.0000		
Gastric ulcer	22 (44.9)	30 (62.5)	0.1044	—	n.s.
Duodenal ulcer	11 (22.4)	8 (16.7)	0.6102		
Solid cancer	5 (10.2)	1 (2.1)	0.2041	—	n.s.
Others (Mallory–Weiss, esophagitis, angiodysplasia, etc.)	9 (18.4)	7 (14.6)	0.7854		
Medications					
Antiplatelets	7 (14.3)	6 (12.5)	1.0000		
Anticoagulants	4 (8.2)	4 (8.3)	1.0000		
NSAIDs	7 (14.3)	8 (16.7)	0.7854		
Proton pump inhibitor	15 (30.6)	2 (4.2)	0.0009	10.147 (2.174-47.358)	0.003
H_2_ receptor blocker	3 (6.1)	5 (10.4)	0.4865		
Comorbid illness					
Hypertension	26 (53.1)	20 (41.7)	0.3115		
Diabetes mellitus	10 (20.4)	11 (22.9)	0.8092		
Hyperlipidemia	7 (14.3)	7 (14.6)	1.0000		
Cerebrovascular diseases	5 (10.2)	7 (14.6)	0.5529		
Cardiovascular diseases	9 (18.4)	5 (10.4)	0.3873		
Liver diseases	2 (4.1)	6 (12.5)	0.1591	—	n.s.
Renal diseases	6 (12.2)	3 (6.3)	0.4865		
Dementia	2 (4.1)	2 (4.2)	1.0000		
Nongastrointestinal malignancies	9 (18.4)	10 (20.8)	0.8026		

**Table 1b:** (b) Clinical characteristics of 45 patients over 75 years old enrolled in this study

Characteristics	Bleeding severity		Univariate *P* value	Multivariate	
	Mild *n* = 27 (%)	Moderate/severe *n* = 18 (%)		OR (95% CI)	*P* value
Age	82.3 ± 1.0	82.2 ± 1.3	0.9587		
Male	15 (55.6)	11 (61.1)	0.7660		
Etiology of bleeding					
Esophageal ulcer	2 (7.4)	0 (0.0)	0.5091		
Gastric ulcer	10 (37.0)	11 (61.1)	0.1376	—	n.s.
Duodenal ulcer	5 (18.5)	2 (11.1)	0.6844		
Solid cancer	5 (18.5)	1 (5.6)	0.3773		
Others (Mallory–Weiss, esophagitis, angiodysplasia, etc.)	5 (18.5)	4 (22.2)	1.0000		
Medications					
Antiplatelets	6 (22.2)	4 (22.2)	1.0000		
Anticoagulants	3 (11.1)	3 (16.7)	0.6703		
NSAIDs	5 (18.5)	2 (11.1)	0.6844		
Proton pump inhibitor	10 (37.0)	1 (5.6)	0.0307	10.000 (1.150-8951)	0.037
H_2_ receptor blocker	3 (11.1)	3 (16.7)	0.6703		
Comorbid illness					
Hypertension	19 (70.4)	12 (66.7)	0.7668		
Diabetes mellitus	6 (22.2)	7 (38.9)	0.3172		
Hyperlipidemia	5 (18.5)	5 (27.8)	1.0000		
Cerebrovascular diseases	5 (18.5)	3 (16.7)	1.0000		
Cardiovascular diseases	7 (25.9)	3 (16.7)	0.7161		
Liver diseases	2 (7.4)	1 (5.6)	1.0000		
Renal diseases	5 (18.5)	2 (11.1)	0.6844		
Dementia	2 (7.4)	1 (5.6)	1.0000		
Nongastrointestinal malignancies	3 (11.1)	4 (22.2)	0.4122		

**Table 1c:** (c) Clinical characteristics of 52 patients younger than 75 years old enrolled in this study

Characteristics	Bleeding severity		Univariate *P* value	Multivariate	
	Mild *n* = 22 (%)	Moderate/severe *n* = 30 (%)		OR (95% CI)	*P* value
Age	62.1 ± 1.8	62.5 ± 2.1	0.4869		
Male	16 (72.7)	20 (66.7)	0.7646		
Etiology of bleeding					
Esophageal ulcer	0 (0.0)	2 (6.7)	0.5023		
Gastric ulcer	12 (54.5)	19 (63.3)	0.5764		
Duodenal ulcer	6 (27.2)	6 (20.0)	0.7402		
Solid cancer	0 (0.0)	0 (0.0)	1.0000		
Others (Mallory–Weiss, esophagitis, angiodysplasia, etc.)	4 (18.2)	3 (10.0)	0.4385		
Medications					
Antiplatelets	1 (4.5)	2 (6.7)	1.0000		
Anticoagulants	1 (4.5)	1 (3.3)	1.0000		
NSAIDs	2 (9.1)	6 (20.0)	0.4420		
Proton pump inhibitor	5 (22.7)	1 (3.3)	0.0716	—	n.s.
H_2_ receptor blocker	0 (0.0)	2 (6.7)	0.5023		
Comorbid illness					
Hypertension	7 (31.8)	8 (26.7)	0.7618		
Diabetes mellitus	4 (18.2)	4 (13.3)	0.7084		
Hyperlipidemia	2 (9.1)	2 (6.7)	1.0000		
Cerebrovascular diseases	0 (0.0)	4 (13.3)	0.1282	—	n.s.
Cardiovascular diseases	2 (9.1)	2 (6.7)	1.0000		
Liver diseases	2 (9.1)	3 (10.0)	1.0000		
Renal diseases	3 (13.6)	3 (6.3)	0.6890		
Dementia	0 (0.0)	1 (3.3)	1.0000		
Nongastrointestinal malignancies	2 (9.1)	7 (23.3)	0.2720		

Values were estimated by Fisher's exact probability test for univariate analysis, and multivariable logistic regression for multivariable analysis. NSAIDs: nonsteroidal anti-inflammatory drugs.

## Data Availability

The data used to support the findings of this study are available from the corresponding author YN on request.
